# Association Between Urinary Phthalates and Pubertal Timing in Chinese Adolescents

**DOI:** 10.2188/jea.JE20140205

**Published:** 2015-09-05

**Authors:** Huijing Shi, Yang Cao, Qing Shen, Yan Zhao, Zhe Zhang, Yunhui Zhang

**Affiliations:** 1 School of Public Health, Fudan University & Key Laboratory of Public Health Safety, Chinese Ministry of Education, Shanghai, China; 2 Division of Epidemiology, Institute of Environmental Medicine, Karolinska Institute, Stockholm, Sweden; 3 Department of Public Health, Karolinska Institutet, Stockholm, Sweden

**Keywords:** phthalate, sexual development, puberty onset, adolescent, body fat

## Abstract

**Background:**

Phthalates are synthetic chemicals and ubiquitous environmental contaminants, with hormonal activity that may alter the course of pubertal development in children.

**Objectives:**

To determine whether exposure to phthalate metabolites is associated with timing of pubertal development in a cross-sectional study of a school-based clustered sample of 503 children from a suburban district in Shanghai, China, who were 7–14 years of age at enrollment (2010 October to November).

**Methods:**

We analyzed six phthalate metabolites in urine samples by isotope-dilution liquid chromatography tandem mass spectrometry. The associations of exposures to phthalates with pubertal timing of testes, breast, and pubic hair development (represented as Tanner stages) were evaluated using an ordered logistic regression model adjusted for chronological age, body fat proportion (BF%), and parental education.

**Results:**

In boys, urinary mono-n-butyl phthalate (MBP) levels were negatively associated with testicular volume, and mono (2-ethyl-5-hydroxyhexyl) phthalate (MEHHP) and mono (2-ethyl-5-oxohexyl) phthalate (MEOHP) levels were negatively associated with pubic hair stages. The odds of being in an advanced stage were decreased by 43%–51%. In girls, mono (2-ethylhexyl) phthalate (MEHP), MEHHP, and MEOHP levels, as well as the sum of these levels, were positively associated with breast stages, and the association was much stronger in girls with high BF%; the odds of being in an advanced stage were increase by 29% to 50%.

**Conclusions:**

Phthalate metabolites investigated in this study show significant associations with pubertal timing both in boys and in girls, especially among girls with high BF%.

## INTRODUCTION

Puberty entails an individual’s transition period from a non-reproductive to a reproductive state and is characterized by rapid physiological changes. The onset of puberty varies by 4 to 5 years among normal boys and girls.^1^ Over the past 50 years, a trend toward earlier age at onset of puberty, especially in girls, has been reported.^2^^–^^6^ At the population level, a secular trend in the timing of puberty may influence behavioral disorders and adult health, which may lead to adverse social and medical conditions.^7^^,^^8^ Although some have thought this trend to be due to improvements in general health and nutrition,^9^ there has also been considerable concern that hormonally active substances, called endocrine-disrupting chemicals (EDCs), are involved in alterations of the onset and progression of pubertal development.^10^^,^^11^ EDCs have been implicated in numerous physiological processes affecting normal reproductive health in human beings and animals.^12^ Specific EDCs that behave like estradiol, such as bisphenol A, may act as hormone agonists and accelerate pubertal development in animal models. However, some EDCs, including phthalates, have both agonist and antagonist effects in animals; these varying effects are likely related to alternative mechanism, dose levels, and exposure timing.^13^

Phthalates are widely used in personal care and consumer products, including children’s toys and medical devices, to make them soft and flexible, as well as in cosmetics, where phthalates act as a vehicle for fragrance.^14^^,^^15^ Humans can be exposed to phthalates through inhalation, ingestion, dermal exposure, and medical treatments.^16^^,^^17^ Previous studies have mainly focused on their adverse effects on male reproductive development.^18^^–^^20^ Recently, human epidemiological studies of the association between phthalate exposure and pubertal development have caused concern. In boys, phthalate exposure has been associated with earlier age at pubarche^21^ and pubertal gynecomastia.^22^ High phthalate exposure has also been associated with changes in pubertal timing in girls,^23^^–^^26^ although this association is controversial.^21^^,^^27^

The effects of phthalates on the timing of puberty, while subtle, may have significant public health implications. A population shift in pubertal timing may confer health risks for later disease, both physical (eg, adult obesity and height loss) and psychological (eg, delinquency, substance use, and risky sexual behavior). Altered onset of puberty and growth in children has been considered a problematic issue in many countries.^28^^–^^30^ However, given the inconsistent results existing among girls and the paucity of evidence in boys, further investigation of the relationship between phthalates exposure and pubertal development in children is urgently needed.

Based on a large cross-sectional survey on pubertal timing and health effects in China, we analyzed urinary phthalate concentrations in a random sample and measured the pubertal stage of children. We aimed to provide further insight into the possible role of phthalate exposure in the timing of pubertal development during a critical time window in children. The hypothesis of our study was that high urinary phthalate concentrations were positively associated with relatively earlier development of pubertal indices in girls but negatively associated with the similar indices in boys.

## MATERIALS AND METHODS

### Study population

After the multi-centered Chinese Puberty Research Collaboration was initiated in September 2010, a large cross-sectional survey—the Pubertal Timing and Health Effects in Chinese Children (PTHEC) study—was conducted in eight cities from October to November 2010.^30^^,^^31^ Shanghai was one of the cities involved in the PTHEC study. By a stratified multistage cluster sampling method, one urban district and one suburban district were selected from eight central urban districts and nine suburban districts in Shanghai. After excluding students with congenital malformations and genetic, metabolic, or chronic endocrine diseases, 3462 students (1787 boys and 1675 girls) aged 6 to 18 years were invited to join the study, which included anthropometric measurement, sexual maturation assessment, and a questionnaire interview. Of these, primary school students in grades three through seven from one suburban district were selected for further laboratory analysis of urine samples. The study was approved by the Ethics Review Committee of Fudan University (IRB#2010-11-0242; 2011-03-0280). Informed consent was explained to all children and their parents, and signed informed consent forms were voluntarily obtained from the parents before participation.

### Data collection

A set of questionnaires was completed by the students and their guardians, which included perinatal factors, demographic variables, perceived physical growth and development, emotion and feeling, physical activities, parental information, sleeping and study habits, dietary habits and intake, and experience of spermarche (in boys) or menarche (in girls).

### Physical examination

Anthropometric measures, including body weight, height, body mass index (BMI), and tricep and subscapular skinfold thicknesses, were measured by physical examination according to WHO-recommended methods and protocols at the time when the urine phthalate samples were collected in all children.^32^ The equipment was calibrated daily using the manufacturer’s calibrator. Body fat proportion (BF%) was calculated using Yao’s formula, which is widely used in Chinese school-age children aged 7–12 years.^33^ The formulae were BF% = 6.931 + 0.428X and BF% = 7.896 + 0.458X for boys and girls, respectively, where X is the sum of tricep and subscapular skin fold thickness in millimeters.

The sexual maturity of testes (in boys), breasts (in girls),^34^ and pubic hair (in both boys and girls) was assessed privately by a male urologist (for boys) or a female pediatrician (for girls). Testicular volume (TV) was estimated by palpation to the nearest whole milliliter using Prader’s orchidometer and divided into four levels (T1–T4) as <4 mL, 4–11 mL, 12–19 mL, and ≥20 mL.^11^^,^^34^^,^^35^ In cases of discrepancy between the left and right side, the largest measurement was used for classification. Sexual maturity stages from stage 1 (indicating immaturity) to stage 5 (indicating full maturity) of breasts (B1–B5) and pubic hair (PH1–PH5) were assessed by inspection and palpation according to the methods of Marshall & Tanner.^36^^,^^37^ The students were asked whether or not they had had their first nocturnal emission (for boys) or first menstrual bleeding (for girls).

### Urinary biomarker measurement

Spot urinary samples were collected from each student on the day of physical examination. All specimens were collected with glass devices to avoid contamination and stored at −20°C until analysis. Six phthalate metabolites were measured: mono-n-butyl phthalate (MBP), mono-methyl phthalate (MMP), monoethyl phthalate (MEP), mono (2-ethylhexyl) phthalate (MEHP), mono (2-ethyl-5-hydroxyhexyl) phthalate (MEHHP), and mono (2-ethyl-5-oxohexyl) phthalate (MEOHP). The sum of MEHP, MEHHP, and MEOHP concentrations was represented as ΣMEHP. Phthalate metabolites in urine extract were resolved using an Agilent 1100 Series high-performance liquid chromatography system (Agilent Technologies, Santa Clara, CA, USA) and detected with an API 2000 electrospray triple quadrupole mass spectrometer (Applied Biosystems, Foster City, CA, USA).^13^ C_4_-labeled internal standards and conjugated internal standards were used to increase the precision of the measurements. Analysts at the Key Laboratory of Public Health Safety, who performed the tests for the present study, were blinded to all information concerning our subjects. For concentrations below the limits of detection (LODs), corresponding to 0.25 µg/L (MMP) and 0.50 µg/L (MBP, MEP, MEHP, MEOHP, and MEHHP), we used an imputed value equal to one-half the LOD.

We used specific gravity (SG) to correct for urinary dilution, as recommended by Hauser et al.^15^ SG was measured using a handheld refractometer (PAL10-S; Atago, Tokyo, Japan). The correction formula was Pc = P × (1.024 − 1)/(SG − 1), where Pc is the specific gravity-corrected phthalate metabolite concentration (µg/L) and P is the experimental phthalate metabolite concentration.

### Statistical methods

Arithmetic mean and standard deviation (SD) of age, height, weight, and BF%, and geometric mean (GM) and 95% confidence interval (CI) of SG-corrected urine phthalate metabolite concentrations were separately calculated for boys and girls. The differences in demographic characteristics and phthalate metabolite levels between boys and girls were tested using a *t*-test or Wilcoxon’s rank-sum test. The correlations between phthalate metabolite levels and covariates were measured using Spearman correlation coefficient *r_s_*.

To evaluate the association of phthalate metabolite levels with pubertal timing, an ordered logistic regression model was constructed to assess the association of sexual maturation of TV, breasts, or pubic hair and SG-corrected urine phthalate metabolite concentrations, adjusting for pre-determined covariates, including chronological age (the number of the months after birth divided by 12), BF%, and parental education. The purpose of this analysis was to compare the pubertal development stage of an adolescent in relation to peers of the same age and BF%. That is, at a given age and BF%, a higher sexual development stage signifies relatively earlier pubertal timing. Because parental education is an important proxy of socioeconomic status of a family and has close relationship with child health and puberty, we included it as a covariate in the model.^38^^,^^39^

Because the SG-corrected phthalate concentrations were approximately log-normally distributed, a natural log transformation was applied to normalize the data. In ordered logistic regression, the predicted probabilities for each TV level and breast or pubic hair stage at different phthalate concentrations were calculated and plotted to illustrate the trend of these probabilities with increased phthalate concentrations.

Because adipose tissue is a source of pubertal hormones,^40^ we also investigated modification of biomarker associations by BF%. We divided the BF% into low BF (BF% ≤20% in boys or ≤25% in girls) and high BF (BF% >20% in boys or >25% in girls) groups and phthalate concentrations into low exposure (≤median concentration) and high exposure (>median concentration) groups, then introduced an interaction term of BF and exposure in the model.

For multivariate analysis, the list-wise deletion method was used for handling missing data, and students were excluded from analyses if any single value for dependent or independent variables was missing.^41^ The difference in demographic characteristics between excluded and included students was compared using the *t* test or Chi-square test.

To assess the robustness of our results to various methodological decisions, we conducted several sensitivity analyses. First, we defined phthalate concentrations as continuous and ordinal variables with two, three, four, and five categories divided by median, tertiles, quartiles, and quintiles, respectively. Second, we fitted univariate models separately for continuous and ordinal phthalate concentration variables to estimate the crude odds ratios (ORs). Third, we introduced other covariates in the univariate models of continuous and ordinal phthalate concentration variables to estimate the adjusted ORs. The goodness of fit of different modeling strategies and ORs was compared across the models.

All tests were two-sided, and a *P*-value of less than 0.05 was considered statistically significant. All statistical analyses were performed with the Stata software version 12.1 (StataCorp, College Station, TX, USA).

## RESULTS

**Figure 1.**
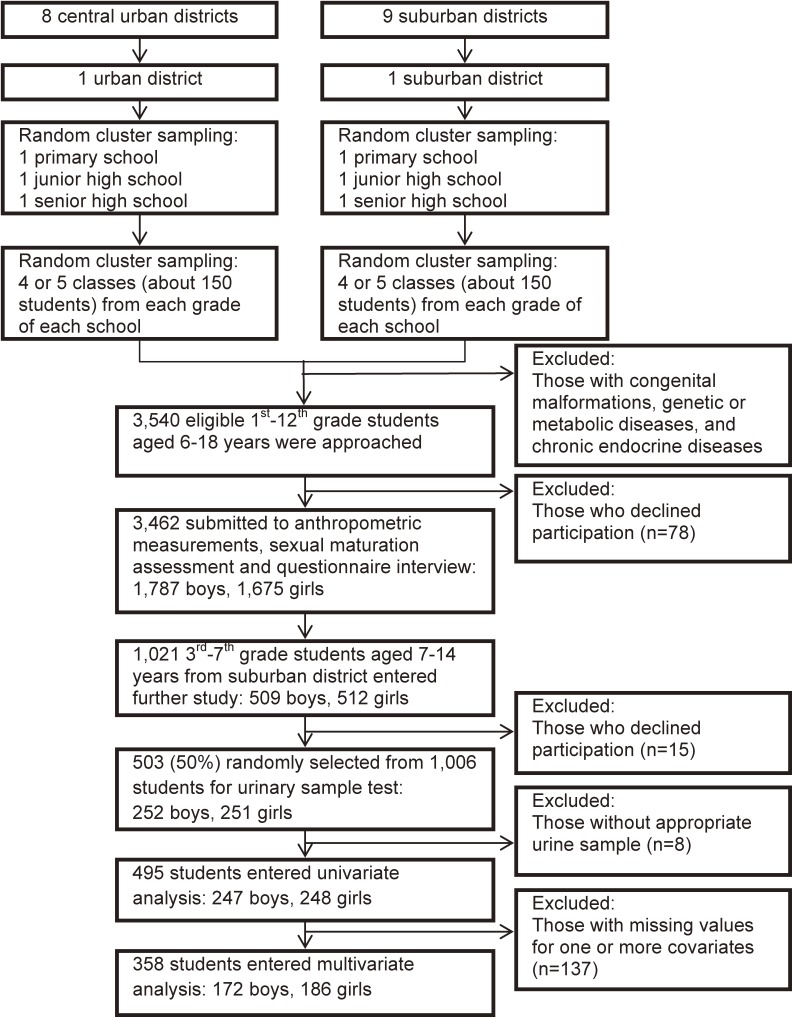
Flow chart of participant recruitment.

Ultimately, 503 primary-school students (252 boys and 251 girls) from a suburban district in Shanghai were selected for urine sample analysis. The detailed flow diagram of recruitment is shown in Figure 1. Due to lack of a qualified urine sample, eight children were excluded from laboratory testing. All studied phthalate metabolites could be detected in all analyzed samples, and the detection rates of mono-phthalates were all higher than 95%, except for MEP and MEHP.

For demographics and exposure characteristics, boys had significantly higher weight and BF%, as well as higher exposure to MMP, MEOHP, and ΣMEHP than girls (Table 1). Over 60% of boys had high BF and over 85% of girls had low BF. The proportion of boys with high phthalate exposure and high BF was over 30%. However, over 40% of girls had low BF and low phthalate exposure, except for exposure to MEHP (see eTable 1). In all children, age at enrollment and parental education were correlated with concentrations of some phthalates, but the relationship was weak (|*r_s_*| < 0.26 and |*r_s_*| < 0.18 for age and parental education, respectively). Except for a weak positive correlation between BF% and MEP concentration (*r_s_* = 0.11, *P* = 0.035), there were no significant correlations between BF% and concentration of other phthalate metabolites.

**Table 1.** Demographic and exposure characteristics

	Boys (*n* = 252)	Girls (*n* = 251)
Mean (SD) age, years	10.7 (1.5)	10.7 (1.5)
Father’s education, *n* (%)		
Primary or lower	1 (0.55)	3 (1.60)
Junior high school	49 (26.78)	37 (19.79)
Senior high school	76 (41.53)	85 (45.45)
College or university	50 (27.32)	59 (31.55)
Graduate education	7 (3.83)	3 (1.60)
Mother’s education, *n* (%)		
Primary or lower	4 (2.20)	2 (1.07)
Junior high school	43 (23.63)	38 (20.32)
Senior high school	71 (39.01)	85 (45.45)
College or university	59 (32.42)	57 (30.48)
Graduate education	5 (2.75)	5 (2.67)
Mean (SD) height, cm	146.1 (11.5)	145.1 (10.9)
Mean (SD) weight, kg^a^	43.8 (14.3)	39.2 (11.5)
Mean (SD) body fat composition, %^a^	24.9 (9.2)	17.7 (7.1)
Boy’s testicular volume, *n* (%)		
<4 mL	137 (54.37)	
4–11 mL	72 (28.57)	
12–19 mL	25 (9.92)	
≥20 mL	18 (7.14)	
Tanner stage of girls’ breast, *n* (%)		
B1		55 (21.91)
B2		74 (29.48)
B3		64 (25.50)
B4		46 (18.33)
B5		12 (4.78)
Tanner stage of pubic hair, *n* (%)^b^		
PH1	219 (86.90)	175 (69.72)
PH2	18 (7.14)	27 (10.76)
PH3	12 (4.76)	41 (16.33)
PH4	3 (1.19)	7 (2.79)
PH5	—	1 (0.40)
Boy’s spermarche/girl’s menarche, *n* (%)		
Yes	8 (4.17)	51 (25.89)
No	184 (95.83)	146 (74.11)
Urinary specific gravity-adjusted phthalate concentrations, µg/L, GM (95% CI)^c^		
MBP	28.57 (25.98, 31.41)	19.60 (16.35, 23.50)
MMP*	14.18 (12.83, 15.67)	11.50 (10.28, 12.87)
MEP	1.15 (0.93, 1.41)	1.14 (0.91, 1.43)
MEHP	1.65 (1.40, 1.94)	1.65 (1.41, 1.92)
MEHHP	16.10 (14.65, 17.68)	14.5 (12.97, 16.22)
MEOHP*	5.77 (5.25, 6.37)	4.72 (4.18, 5.33)
ΣMEHP*	24.49 (22.31, 26.89)	21.52 (19.21, 24.11)

CI, confidence interval; GM, geometric mean; MBP, monobutyl phthalate; MEHHP, mono-(2-ethyl-5-hydroxyhexyl) phthalate; MEHP, mono-(2-ethylhexyl) phthalate; MEOHP, mono-(2-ethyl-5-oxohexyl) phthalate; MEP, mono-ethyl phthalate; MMP, mono-methyl phthalate; ΣMEHP, sum of MEHP, MEHHP, and MEOHP concentrations.

^a^Significant difference between boys and girls, *P* < 0.05, *t* test.

^b^Significant difference between boys and girls, *P* < 0.05, Wilcoxon rank-sum test.

^c^Values based on test results for 247 boys and 248 girls.

The crude ORs derived from univariate ordered logistic regression models show that TV in boys, breast stage in girls, and pubic hair stage in boys and girls were all negatively associated with concentrations of two to four non-MEP phthalate metabolites (data not shown). MEP level was positively associated with increased TV in boys and earlier menarche in girls, and the risk of presenting with larger TV and earlier menarche than age- and BF%-matched peers were increased by 18% and 33%, respectively.

Due to missing data on covariates, 137 children were excluded from multivariate ordered logistic analysis. Analyses revealed no significant differences between excluded and included children in demographic characteristics and phthalate concentrations. After adjusting for chronological age, BF%, and parental education, MBP level was negatively associated with TV, and MEHHP and MEOHP levels were negatively associated with pubic hair stages in boys. The risk of being in advanced stages was decreased by 43%–51% compared to age- and BF%-matched peers. The association between phthalate levels and breast stage in girls was reversed after adjusting for covariates. MEHP, MEHHP, MEOHP, and ΣMEHP were positively associated with breast stage, and the risk of being in advanced stages was increase by 29% to 50% compared to age- and BF%-matched peers (Table 2). No association was found between phthalate levels and spermarche in boys or pubic hair stage and menarche in girls.

**Table 2.** Odds ratios and 95% confidence intervals^a^ of presenting higher versus lower pubertal development levels per 1-unit increase in logarithmic urine specific gravity-adjusted phthalate concentrations

Boys	Testicle volume (*n* = 176)	Pubic hair (*n* = 176)	First nocturnal emission (*n* = 170)
MBP	0.58 (0.34, 0.99)*	0.91 (0.45, 1.83)	1.31 (0.43, 3.98)
MMP	1.46 (0.94, 2.26)	0.74 (0.43, 1.27)	1.38 (0.52, 3.66)
MEP	1.10 (0.90, 1.35)	1.15 (0.89, 1.48)	0.87 (0.53, 1.41)
MEHP	1.23 (0.94, 1.62)	0.87 (0.61, 1.25)	0.77 (0.42, 1.43)
MEHHP	0.68 (0.41, 1.11)	0.52 (0.28, 0.97)*	1.05 (0.36, 3.05)
MEOHP	0.72 (0.44, 1.15)	0.49 (0.26, 0.91)*	0.93 (0.32, 2.70)
ΣMEHP	0.82 (0.51, 1.33)	0.57 (0.30, 1.07)	1.14 (0.40, 3.29)

Girls	Breast (*n* = 182)	Pubic hair (*n* = 182)	Menarche (*n* = 178)

MBP	1.13 (0.94, 1.35)	1.10 (0.87, 1.40)	0.91 (0.68, 1.23)
MMP	0.83 (0.59, 1.15)	0.96 (0.62, 1.50)	1.10 (0.61, 1.97)
MEP	1.00 (0.86, 1.17)	1.08 (0.90, 1.31)	1.11 (0.89, 1.39)
MEHP	1.29 (1.01, 1.64)*	1.11 (0.82, 1.50)	0.95 (0.64, 1.41)
MEHHP	1.45 (1.06, 1.98)*	1.00 (0.68, 1.46)	0.72 (0.44, 1.19)
MEOHP	1.46 (1.09, 1.95)*	1.09 (0.77, 1.56)	0.75 (0.48, 1.20)
ΣMEHP	1.50 (1.10, 2.04)*	1.05 (0.72, 1.54)	0.74 (0.45, 1.23)

MBP, monobutyl phthalate; MEHHP, mono-(2-ethyl-5-hydroxyhexyl) phthalate; MEHP, mono-(2-ethylhexyl) phthalate; MEOHP, mono-(2-ethyl-5-oxohexyl) phthalate; MEP, mono-ethyl phthalate; MMP, mono-methyl phthalate; ΣMEHP, sum of MEHP, MEHHP, and MEOHP concentrations.

^a^Adjusted for chronological age, body fat composition and parental education.

*P < 0.05.

**Figure 2.**
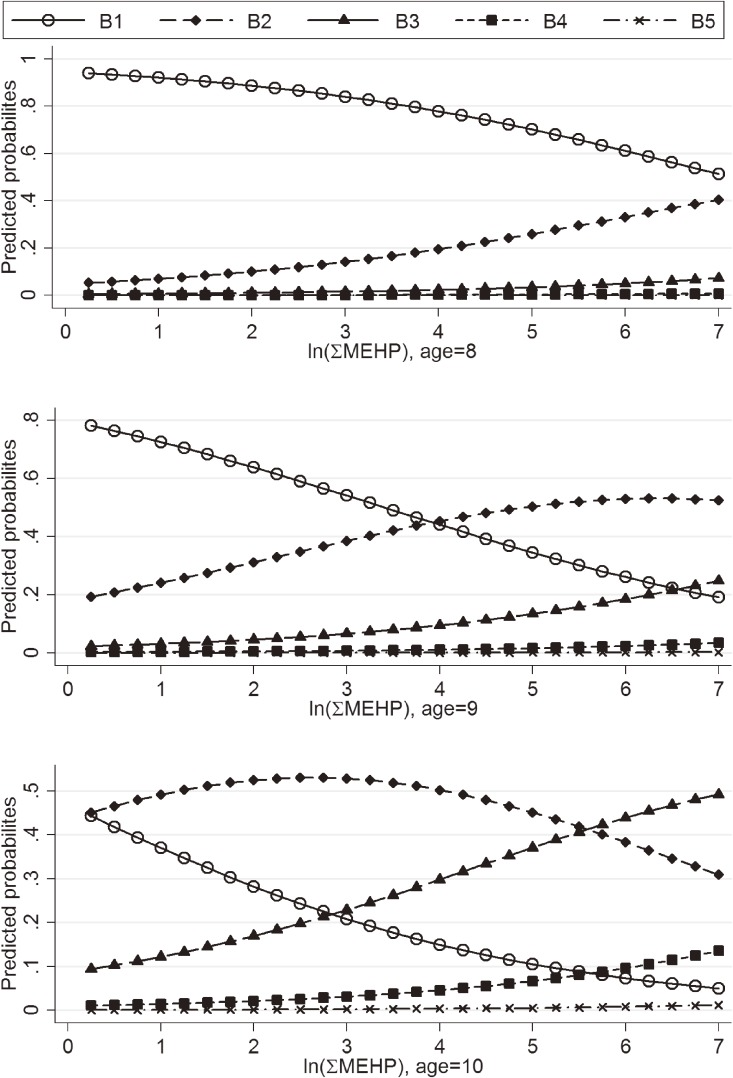
Predicted probabilities of breast stages (B1–B5) of girls (*n* = 182) with ΣMEHP concentrations at ages 8, 9, and 10 years.

The predicted probabilities of having larger TV and higher pubic hair stage in boys decreased with MBP, MEHHP, and MEOHP concentrations (data not shown). The predicted probabilities of presenting with lower breast stage were decreased, and predicted probabilities of presenting higher breast stage were increased, with MEHP, MEHHP, MEOHP, and ΣMEHP concentrations, and this was most apparent in ΣMEHP for older girls (Figure 2).

Although no statistically significant interactions were found between BF% and phthalate levels, we did find that the associations of phthalate concentrations with breast development were much stronger in girls with high BF%. Compared with girls with high phthalate levels and low BF%, the ORs of presenting with higher breast stage in girls with high phthalate levels and high BF% were 3.52 versus 1.31, 6.99 versus 1.16, 7.71 versus 1.42, and 7.83 versus 1.50 for MEHP, MEHHP, MEOHP, and ΣMEHP, respectively (Table 3).

**Table 3.** Association of breast development with interaction between body fat^a^ and phthalate concentrations^b^ in girls

Exposures	OR	95% CI	*P*^c^
MEHP			
low PC, Low BF	Reference		
low PC, High BF	2.76	1.16, 6.60	0.022
high PC, Low BF	1.31	0.71, 2.41	0.391
high PC, High BF	3.52	0.45, 27.48	0.231

MEHHP			
low PC, Low BF	Reference		
low PC, High BF	2.30	0.97, 5.45	0.058
high PC, Low BF	1.16	0.63, 2.14	0.624
high PC, High BF	6.99	1.03, 47.29	0.046

MEOHP			
low PC, Low BF	Reference		
low PC, High BF	2.51	1.06, 5.99	0.037
high PC, Low BF	1.42	0.78, 2.61	0.254
high PC, High BF	7.71	1.13, 52.52	0.037

ΣMEHP			
low PC, Low BF	Reference		
low PC, High BF	2.57	1.08, 6.10	0.033
high PC, Low BF	1.50	0.81, 2.75	0.195
high PC, High BF	7.83	1.15, 53.32	0.035

BF, body fat; CI, confidence interval; MEHHP, mono-(2-ethyl-5-hydroxyhexyl) phthalate; MEHP, mono-(2-ethylhexyl) phthalate; MEOHP, mono-(2-ethyl-5-oxohexyl) phthalate; OR, odds ratio; PC, phthalate concentration; ΣMEHP, sum of MEHP, MEHHP, and MEOHP concentrations.

^a^Divided into low and high groups by body fat composition ≤25% and >25% for girls.

^b^Divided into low exposure and high exposure groups by ≤median and >median of specific phthalate concentrations of all children.

^c^Adjusted for chronological age and parental education.

## DISCUSSION

In a cross-sectional analysis of a subgroup of the large multi-centered PTHEC study, we examined the associations between concurrent exposure to phthalates, which are known to possess hormonal activity, and pubertal development. The detection rates of the examined urinary phthalate metabolites were all over 80%, which indicated that exposure to phthalates was a routine occurrence for children. The average concentrations of phthalate metabolites ranged from to 1.14 to 28.57 µg/L. These levels were comparable with those reported by Wang et al^42^; however, urinary concentrations of phthalate metabolites in our study were significantly higher in boys than in girls.

Consistent with our a priori hypothesis, positive trends were observed for the association of phthalate biomarkers with breast development in females. Specifically, MEHP, MEHHP, MEOHP, and ΣMEHP were found to be associated with 29% to 50% increases in the risk of being in an advanced breast stages at a given age. These results were consistent with previous findings,^23^^,^^24^^,^^43^ which suggested that high phthalate exposure might be one cause of premature breast development. However, in one American study, Lomenick et al reported no difference in phthalate exposure in girls with and without precocious puberty.^25^ In several Danish studies, Frederiksen et al did not find any association between phthalate exposure and breast development.^21^^,^^26^ Furthermore, in a study by Wolff et al, low-molecular-weight phthalate biomarkers were found to have a weak positive association with breast stage, while high-molecular-weight phthalate metabolites had no association.^22^ These discrepancies might be due to differences in the levels of phthalates measured in different studies. The subjects in our study were from a randomized cluster sample with an age range (from pre-puberty to the early and middle periods of puberty) that involves relatively larger interpersonal variation in breast development timing, which increased the representativeness of the samples and might more comprehensively and sensitively reflect the association between environmental exposure and varying pubertal timing. The inverse association found between phthalate exposure and girls’ breast development, even after adjusting for confounding variables, may be due to the interaction between phthalate metabolites and body fat. Because of the liposolubility of phthalates, the effect of phthalates on breast development would be more evident in girls with more body fat, who might have greater phthalate accumulation in the body; such a relationship was confirmed in the interaction analysis.

The effect of phthalate exposure on male pubertal development has been sparsely evaluated. Mieritz et al did not find any association between current phthalate exposure and pubertal timing,^44^ while higher di-n-butyl phthalate exposure was found to be associated with earlier age at pubarche in another study.^26^ However, in the present study, MBP was found to be associated with a 42% decrease in the risk of larger TV, and MEHHP and MEOHP were found to be associated with 48% to 51% decreases in the risk of being in advanced pubic hair stages, even after controlling for some potential confounders. Our results suggest negative associations between phthalate exposure and pubertal development in males.

Pubertal onset and progression is primarily regulated by the endocrine system through chemical messengers, specifically the sexual hormones. Both animal and human findings suggest that phthalates have antiandrogenic properties.^45^^–^^48^ In males, androgen levels determine the onset of testicular growth and pubic hair development, so we thought that delayed pubertal development in males in our study might be attributed to the antiandrogenic effects of phthalates. However, one of the etiologic explanations for advanced breast development in females is increased estrogenic sensitivity or estrogen-androgen ratio in breast tissue, or both. As in vivo and in vitro studies have indicated negligible estrogenic activity for DEHP,^49^ we speculated that the anti-androgenic effects of DEHP metabolites (MEHP, MEHHP, and MEOHP) resulted in altered estrogen-androgen balance and influenced breast tissue responsiveness to estrogen.

Phthalates, which are considered environmental obesogens, tend to accumulate in fat tissues. Phthalate exposure might contribute to adipogenesis and induce obesity. Low levels of MEHP have been shown to promote adipocyte differentiation in a dose-dependent manner in mice.^50^ Urinary phthalate levels have also been shown to be associated with body size indices in humans.^42^ In the present study, although no statistically significant interactions were found between BF% and phthalate concentrations, the association between phthalate levels and breast development was found to be much stronger in girls with high BF%, which indicates the complexity of phthalates’ impact on adipose tissue biology, hormone systems, and the central hypothalamic-pituitary-gonadal axis, than in those with low BF%.^25^^,^^26^^,^^50^ Follow-up studies on sex hormone levels and pubertal timing are needed to further explore the interactions among phthalate exposure, BF%, and puberty onset in children.

The sensitivity analyses indicated that, when we included the phthalate concentrations as ordinal variables in the model, the association between phthalate levels and pubertal development indices disappeared or reversed with decreased ordinal categories of phthalate concentrations. The most consistent results were found between the models using five-category ordinal phthalate concentrations and the models using continuous phthalate concentrations, which suggests that treating phthalate concentrations as continuous variables would be more suitable for risk assessment purposes.

There are some limitations to our study. First, we used spot urine samples to assess each subject’s phthalate levels. Due to the short half-lives of phthalate metabolites, a single measurement of spot urine samples might not perfectly represent long-term exposure. Second, we did not measure circulatory levels of sex hormones. Although associations between phthalate concentrations and pubertal timing both in boys and in girls were found in the current study, we could not determine whether or not the associations are the result of hormonal activities of phthalate metabolites. Third, pubertal timing could determine some behaviors associated with phthalate exposures, such as eating packaged foods. Although we investigated dietary habits and intakes in the questionnaire, diet and behavior bias could not be excluded. Furthermore, the potential confounding effects of birth outcomes, living environment in young childhood, or exposure to other chemicals, especially chemicals with direct or indirect antiandrogenic properties, is unclear and cannot be accurately assessed.

To our knowledge, this is the largest study to date to investigate the association between phthalate concentrations and pubertal development in a community sample of primary-school students. In addition, our study used a detailed questionnaire survey and physical examination by trained specialists, which minimized the potential for misclassification. Furthermore, ordered logistic regression, an extension of logistic regression, was used in our study, which is particularly appropriate to investigate the relationship of ordered outcomes with demographic characteristics, biochemistry data, and self-assessment of health. The results from ordered logistics regression models can be more valid and sometimes more informative than those of ordinary least-squares regression models when the distribution of outcome is highly non-normal.^51^^,^^52^

In conclusion, we found significant associations between urinary phthalate metabolite concentrations and pubertal stages in children aged 7 to 14 years. High phthalate concentrations were associated with delayed pubertal development in boys and advanced pubertal development in girls, which might reflect the antiandrogenic action of phthalates.

## ONLINE ONLY MATERIAL

eTable 1. Distribution of low and high body fat (BF)^a^, and low and high phthalate exposure.
