# Supplementary material for: Association Between Urinary Phthalates and Pubertal Timing in Chinese Adolescents
Source: J Epidemiol. 2015 Sep 5;25(9):574–82. doi: 10.2188/jea.JE20140205 (PMC4549609; doi:10.2188/jea.JE20140205)
Supplement: eTable 1. [file je-25-574-s001.pdf]

eTable 1. Distribution of low and high body fat[a] and low and high exposure of phthalates[b]

	Boys, n (%)	Girls, n (%)
MBP		
low MBP, low BF	46 (18.62)	110 (44.35)
low MBP, high BF	71 (28.74)	21 (8.47)
high MBP, low BF	51 (20.65)	104 (41.94)
high MBP, high BF	79 (31.98)	13 (5.24)
MMP		
low MMP, low BF	39 (15.79)	117 (47.18)
low MMP, high BF	65 (26.32)	26 (10.48)
high MMP, low BF	58 (23.48)	97 (39.11)
high MMP, high BF	85 (34.41)	8 (3.23)
MEP		
low MEP, low BF	57 (23.08)	109 (43.95)
low MEP, high BF	62 (25.10)	19 (7.66)
high MEP, low BF	40 (16.19)	105 (42.34)
high MEP, high BF	88 (35.63)	15 (6.05)
MEHP		
low MEHP, low BF	49 (19.84)	92 (37.10)
low MEHP, high BF	77 (31.17)	29 (11.69)
high MEHP, low BF	48 (19.43)	122 (49.19)
high MEHP, high BF	73 (29.55)	5 (2.02)
MEHHP		
low MEHHP, low BF	47 (19.03)	109 (43.95)
low MEHHP, high BF	65 (26.32)	27 (10.89)
high MEHHP, low BF	50 (20.24)	105 (42.34)
high MEHHP, high BF	85 (34.41)	7 (2.82)
MEOHP		
low MEOHP, low BF	46 (18.62)	114 (45.97)
low MEOHP, high BF	61 (24.70)	27 (10.89)
high MEOHP, low BF	51 (20.65)	100 (40.32)
high MEOHP, high BF	89 (36.03)	7 (2.82)
ΣMEHP		
low ΣMEHP, low BF	49 (19.84)	106 (42.74)
low ΣMEHP, high BF	66 (26.72)	27 (10.89)
high ΣMEHP, low BF	48 (19.43)	108 (43.55)
high ΣMEHP, high BF	84 (34.01)	7 (2.82)

BF, body fat; MBP, monobutyl phthalate; MEHHP, mono-(2-ethyl-5-hydroxyhexyl) phthalate; MEHP, mono-(2-ethylhexyl) phthalate; MEOHP, mono-(2-ethyl-5-oxohexyl) phthalate; MEP, mono-ethyl phthalate; ΣMEHP, sum of MEHP, MEHHP, and MEOHP concentrations.

[a] Divided into low BF and high BF groups by body fat composition ≤20% and >20% for boys, and body fat ≤25% and >25% for girls, respectively

[b] Divided into low exposure and high exposure groups by phthalate concentrations ≤ or > respective medians of all children
